# Instant killing of pathogenic chytrid fungi by disposable nitrile gloves prevents disease transmission between amphibians

**DOI:** 10.1371/journal.pone.0241048

**Published:** 2020-10-29

**Authors:** Valarie Thomas, Pascale Van Rooij, Celine Meerpoel, Gwij Stegen, Jella Wauters, Lynn Vanhaecke, An Martel, Frank Pasmans

**Affiliations:** 1 Wildlife Health Ghent, Department of Pathology, Bacteriology and Avian Diseases, Faculty of Veterinary Medicine, Ghent University, Merelbeke, Belgium; 2 Laboratory of Chemical Analysis, Faculty of Veterinary Medicine, Ghent University, Merelbeke, Belgium; University of South Dakota, UNITED STATES

## Abstract

To prevent transmission of the pathogenic chytrid fungi *Batrachochytrium dendrobatidis* (*Bd*) and *Batrachochytrium salamandrivorans* (*Bsal*), hygiene protocols prescribe the single use of disposable gloves for handling amphibians. We discovered that rinse water from nitrile gloves instantly kills 99% of *Bd* and *Bsal* zoospores. Transmission experiments using midwife toads (*Alytes obstetricans*) and *Bd*, and Alpine newts (*Ichthyosaura alpestris*) and *Bsal*, show that the use of the same pair of gloves for 2 subsequent individuals does not result in significant transmission of any chytrid fungus. In contrast, handling infected amphibians bare-handed caused transmission of *Bsal* in 4 out of 10 replicates, but did not result in transmission of *Bd*. Based on the manufacturer’s information, high resolution mass spectrometry (HRMS) and colorimetric tests, calcium lactate and calcium nitrate were identified as compounds with antifungal activity against both *Bd* and *Bsal*. These findings corroborate the importance of wearing gloves as an important sanitary measure in amphibian disease prevention. If the highly recommended single use of gloves is not possible, handling multiple post-metamorphic amphibians with the same pair of nitrile gloves should still be preferred above bare-handed manipulation.

## Introduction

Skin infections caused by the chytrid fungi *Batrachochytrium dendrobatidis* (*Bd*) and *Batrachochytrium salamandrivorans* (*Bsal*) pose a serious threat to amphibian biodiversity [1–3]. While human-mediated spread of these Asian fungi has made *Bd* a cosmopolite, the emergence of Chytridiomycosis due to *Bsal* is hitherto limited to Europe [4–6], where *Bsal* is increasingly reported both in the wild [7–9], and in captive collections [10–12]. In the Netherlands, Germany and Belgium, *Bsal* has caused collapses of several fire salamander populations [7, 8], and it has also been detected in the wild in a marbled newt population in Spain [13]. The possibility of further introduction of hypervirulent *Bd* or *Bsal* strains or yet unknown pathogens poses a continuous threat.

Handling amphibians without due precautions may contribute to disease transmission. The development and correct application of proper hygiene protocols are therefore crucial in minimizing further spread of these pathogens. Hygiene protocols recommend the single use of disposable gloves for handling amphibians [14, 15]. However, when sampling large numbers of animals within a population, this significantly adds to sampling costs, waste and time [14]. The use of gloves, and guidelines governing the use of gloves in amphibian research, were precipitated by the discovery of *Bd* and ranaviruses. These highly virulent pathogens, caused widespread amphibian mortality and extinctions [16] and resulted in advocacy for glove-use when manipulating more than one amphibian, to prevent dissemination of emerging infectious diseases. Despite the use of gloves being recommended to prevent disease transmission, there have also been reports of deleterious effects on amphibians (both adult and juveniles) after contact with latex [17], nitrile and vinyl gloves [18, 19].

The observation of Mendez et al. [14] that nitrile and vinyl gloves exert a killing effect on *Bd* under controlled laboratory conditions, adds considerable weight to the use of disposable gloves [14]. The killing effect was likely to be ascribed to the presence of a powder on the outside of the gloves, coming from the manufacturing process [14], but the precise fungicidal compounds could not be identified. According to the manufacturers’ information, the nitrile gloves we assayed throughout this study are covered at the outside with a coagulant providing the gloves a firm grip (and not intended to have an antimicrobial action). This coagulant layer consists of more than 20 compounds with calcium nitrate, a polystearate compound and water as main compounds.

The lack of and urgent need for *Bsal*-specific hygiene protocols that prevent transmission of *Bsal* in captive and free-ranging environments, prompted us to investigate the efficacy of nitrile gloves against *Bsal*. We first quantified the fungicidal effect of nitrile gloves on *Bsal* and *Bd in vitro* and investigated which major free chemical compounds were present in washes obtained from nitrile gloves. Next, we determined to which extent wearing nitrile gloves prevents transmission of *Bd* between frogs and *Bsal* between salamanders. Transmission rates of *Bd* and *Bsal* were compared between treatments (gloves versus bare-hands) in a non-parametric Mann-Whitney U test. Log odds and transmission probability among newts exposed to source animals with varying *Bsal* infection loads was analysed also, using binomial logistic regression and a one-tailed Fisher Exact Test using R statistical software [20].

## Materials and methods

### *Batrachochytrium spp*. strains and culture conditions

*Bd* isolate JEL 423 was kindly provided by Dr. Joyce Longcore. Quality control consisted of recording the number of passages, visual, qualitative assessment of fungal cultures, absence of bacteria and other contaminants. It was maintained in TGhL-broth at 20°C, following routine methods [21]. To obtain *Bd* zoospores, 2mL of a 5 days old broth culture was inoculated on TGhL agar and incubated for 5 to 7 days at 20°C. Zoospores were harvested by flooding the agar plates with 2mL distilled water.

AMFP 13/1 is the strain type of *B*. *salamandrivorans* and was isolated by the authors [2]. The zoospores were collected from a 7-days old culture (isolate AMFP 13/1, isolated from *Salamandra salamandra*, the Netherlands) maintained on TGhL-broth in a 75cm^2^ cell culture flask at 15°C [2]. If necessary, production and sporulation of sporangia was stimulated by replacing TGhL broth with an equal amount of mPmTG broth (1g peptonized mik, 1g tryptone, 5 glucose in 100ml distilled water). Once mature zoosporangia were observed by microcopy, mPmTG was removed, the culture was flooded with 10 ml water and typically incubated overnight to 24 hours at 15°C till harvesting of the zoospores. Prior to inoculation the zoospores were washed with sterile distilled water, centrifuged 5 minutes at 3000 rpm and counted in lugol with a haemocytometer.

### Sampling of nitrile gloves

Nitrile gloves were sampled for the subsequent investigation of the presence of chemicals with potential fungicidal and fungistatic effects on *Bd* and *Bsal*. Disposable powder-free white nitrile gloves (Eco nitrile PF 250 gloves; ecoSHIELD Scientific, Ede, The Netherlands) were used for all experiments. This type of glove is similar to nitrile gloves used in Europe and worldwide by researchers and amphibian handlers in labs, animal facilities or during monitoring activities. To take into account possible differences between batches, 10 gloves from each of 3 different batches of nitrile gloves were included in each of the 3 experimental set-ups. To sample the active substance(s) present on the outside of the gloves, 1 ml HPLC water was applied onto gloved hands, gloves were shortly rubbed over each other to spread the HPLC water uniformly over the gloves and the rinse water was collected in a sterile plastic recipient. This was repeated 5 times (1 pair of gloves each time) to obtain a large amount of rinse water. Specifics on the nitrile gloves used are presented in S1 and S2 Tables.

Although the used nitrile gloves were indicated powder-free, the obtained rinse water was whitish and opaque. The washes were centrifuged 1 min at 5000rpm and the supernatant was divided in aliquots and stored at-70°C till further use.

### Effect of the rinse water on *Bd* and *Bsal* zoospore motility

Glove rinse water samples were investigated to determine the effects of chemicals detected in the rinse water on the motility of *Bd* and *Bsal*. Impairment of motility of the zoospores is likely to affect dispersal and transmission ability of the fungus. Five μL of a *Bsal* or *Bd* zoospore suspension (each containing 2x10^5^ zoospores/mL) were added to 20μL rinse water in a 96-well plate. The motility of the zoospores was checked immediately under an inverted microscope (Olympus CKX 41, Hamburg, Germany) and was compared to negative control samples (5μL inoculum added to 20μL sterile distilled water). The time lapse to achieve complete immobility of the zoospores in the rinse water was recorded. The assays investigating the effect of the rinse water on *Bd* and *Bsal* motility were performed in triplicate (for each batch of nitrile gloves) with two replicate wells per replication. The mean time to immobility for *Bd* and *Bsal* and standard errors were calculated.

### Effect of the rinse water on *Bd* and *Bsal* zoospore viability and growth

The viability and growth assays were performed to assess fungal viability after exposure to calcium lactate and calcium nitrate. A microorganism’s growth rate is one determinant of its ability to infect a host and cause disease. In the case of *Bsal* and *Bd*, the growth rate will determine the fungal load that hosts are exposed to and the outcome (higher fungal loads are associated with higher probability of disease and mortality). Glove rinse-water samples were investigated to determine whether and to what extent chemicals detected in the rinse-water would affect the viability and growth of *Bd* and *Bsal* zoospores and zoosporangia.

Rinse water collected from the nitrile glove batches, was filter sterilized over a 0.2μm syringe filter (Whatman, GE Healthcare, Diegem, Belgium). In a 96-well plate, 20μL inoculum containing 2x10^5^ *Bd* or *Bsal* zoospores was added to a 1:1 mixture of concentrated TGhL broth (16g tryptone, 2g hydrolysed gelatine, 4g lactose in 500mL distilled water) and rinse water. Negative controls consisted of 20μL inoculum added to a 1:1 mixture of double concentrated TGhL-broth and filter sterilised HPLC water. Plates were sealed and incubated at 15°C (*Bsal*) or 20°C (*Bd*). During 7 days, zoospore motility and growth were checked under an inverted microscope. The assay investigating the effect of rinse water on *Bd* and *Bsal* viability was performed using duplicate wells for rinse water samples from 10 gloves from each batch (3 batches). The assay was performed in triplicate (three independent assays for each pathogen). The mean viability and standard errors, after exposure to the rinse water, were also calculated for *Bd* and *Bsal*.

### Quantification of the fungicidal effect by EMA-qPCR

This assay was performed to determine the number of *Bd* and *Bsal* zoospores that were killed by the rinse water. We then calculated the difference between the initial viable spores added to the rinse water and the number of live cells detected by the EMA-qPCR. The combination of the viable-death stain ethidium monoazide (EMA; Sigma-Aldrich, Bornem, Belgium) and quantitative PCR into one test (EMA-qPCR) [22] was used to quantify the killing activity of the rinse water against *Bd* and *Bsal* zoospores. A 10μL zoospore suspension containing approximately 2x10^5^ live *Bsal* zoospores or 1x10^5^ live *Bd* zoospores was added to 50μL rinse water or HPLC water (negative controls). Positive controls consisted of 10μL heat-killed zoospores (heated at 85°C for 15 min) added to 50μL HPLC water.

Viability after exposure to rinse water, heat and hplc water was also compared among *Bd* and *Bsal* zoospores. The quantification of the fungicidal effect of EMA-qPCR, was done in triplicate (three times) with two replicate wells used for each batch of gloves for three batches. The mean of each batch was also calculated for each of the experiments.

Approximately 15 seconds after addition of the zoospore suspension, the samples were subdivided into 2 equal aliquots. One aliquot was used to enumerate the total number of zoospores in each sample, while the other aliquot was first pre-treated with EMA to assess the number of viable zoospores in each sample. Procedures were as described by Blooi et al. [22]. Because EMA-concentrations for staining *Bd* (25μg/mL) were found to have a negative effect on *Bsal* viability, *Bsal* zoospores were stained with a ten-fold lower concentration (2.5μg/mL).

DNA was extracted in Prepman Ultra Sample Preparation Reagent (Life Technologies, Ghent, Belgium). Samples were diluted 1:10 in HPLC water and were run in duplicate with *Bd* or *Bsal* genomic equivalent (GE) standards of 1000, 100, 10, 1 and 0.1 GE [23, 24]. Samples were considered positive when a sigmoidal amplification occurred and the detection threshold of 0.1 GEs per reaction was exceeded for both duplicate samples. The fungicidal activity of the rinse water was quantified in triplicate for each of the 3 nitrile glove batches. Fungicidal activity was expressed as ‘log (10) viable spores added to the rinse water–log (10) viable spores recovered immediately after addition’.

We evaluated the overall differences in zoospore viability for each variable (rinse water versus heat and hplc water, knowing that the presence or absence of antifungal compounds will affect zoospore viability differently) and differences between batches by using either an ANOVA or the non-parametric Kruskal-Wallis and Mann-Whitney U test. For each variable, an ANOVA model was run. Normality of the residuals for each model was evaluated using a QQ plot and Shapiro-Wilks test. Models without normally distributed residuals were disregarded, and a Kruskal-Wallis or Mann Whitney U test was used instead for those variables. When applicable, a Tukey *post hoc* test was carried out to identify differences between groups or a Welch or Brown-Forsythe *post hoc* test was run when dealing with unequal variances and sample sizes. All statistical analyses were carried out with the SPSS software [25].

### *In vivo* evaluation of the efficacy of nitrile gloves in preventing disease transmission

This assay was performed to investigate whether and to what extent the use of nitrile gloves prevented the transmission of *Bd* and *Bsal* to contact animals from source animals. All *in vivo* experiments complied with ethical and biosecurity standards. The protocol was approved by the Ethical Committee of the Faculty of Veterinary Medicine, Ghent University, Belgium (application number EC2015/30). Because of their respective susceptibility to *Bsal* and *Bd* infection, Alpine newts (*Ichthyosaura alpestris*) [4, 8] and common midwife toads (*Alytes obstetricans*) [26, 27] were chosen as models for studying *Bsal* and *Bd* transmission, respectively. Forty captive bred, sub-adult/adult alpine newts and forty sub-adult/adult midwife toads were involved in this study. All animals were purchased from commercial breeders and swab-sampled upon arrival. They were tested for the presence of *Batrachochytrium spp*. using qPCR [23, 24].

The animals were acclimatised during 2 weeks and were housed in groups of 10 individuals in ventilated plastic containers (620 x 380 x 150mm) lined with moist tissue, provided with terracotta shells as shelter and a water basin; they were fed once weekly calcium and vitamin powdered crickets and mealworms *ad libitum*. Alpine newts were housed at 15°C, while midwife toads were housed at 20°C.

**Fig 1:**
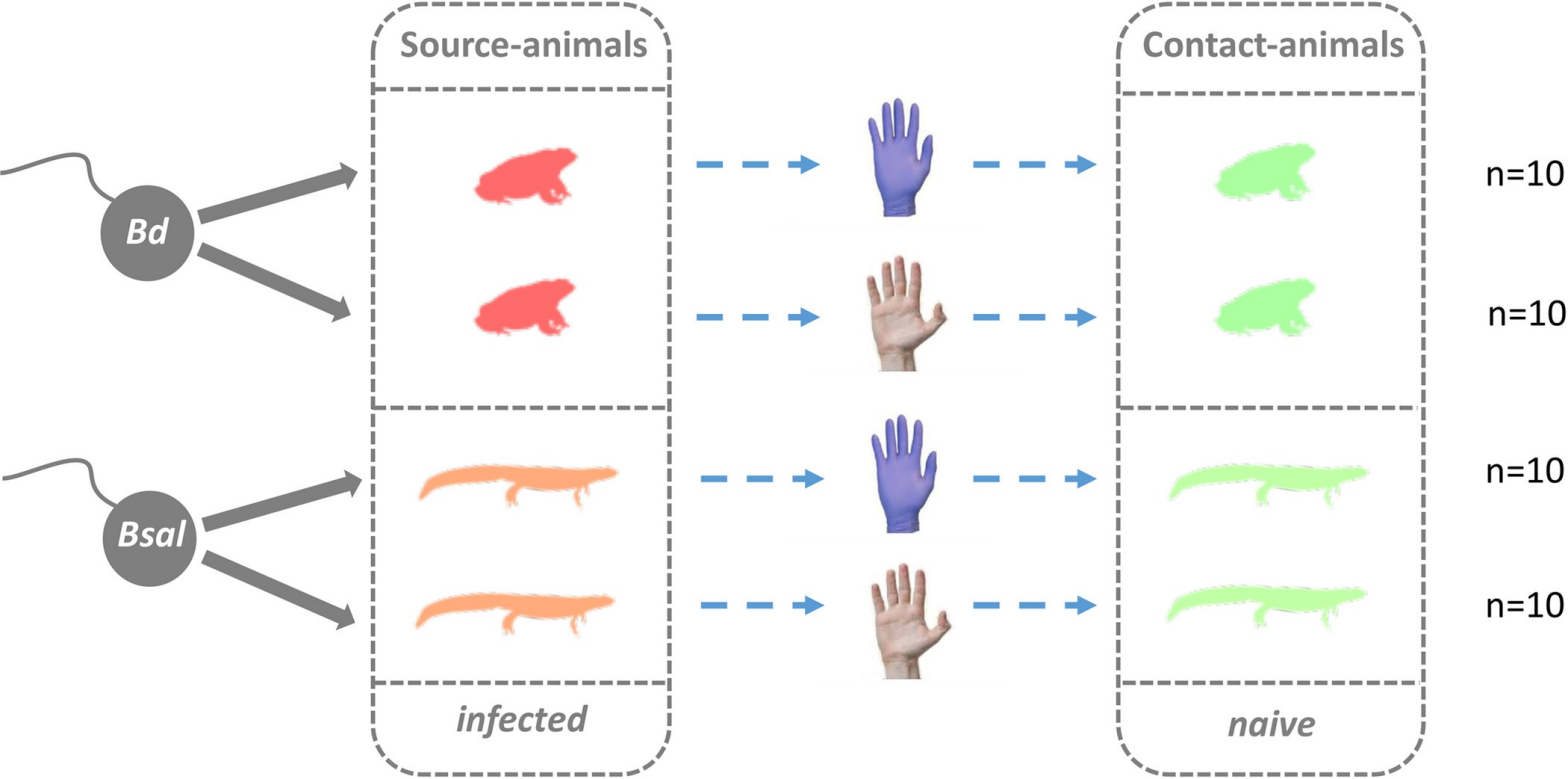
Flowchart of the *in vivo* transmission of *Bd* and *Bsal* via nitrile gloves or bare hands. *Bd*-infected midwife toads (red), *Bsal*-infected alpine newts (orange); naïve toads and newts (green). Each transmission treatment (gloves versus bare hands) was performed in ten animals (n = 10).

As shown in Fig 1, for each model species, 20 individuals were inoculated with *Bd* or *Bsal* and served as ‘source-animals’, while another 20 individuals served as ‘contact-animals’. One source and one contact animal were handled with a pair of gloves. For inoculation, source-animals were individually exposed to 1x10^6^ zoospores of *Bd* (isolate JEL 423) or *Bsal* (isolate AMFP 13/1) during 24 hours, in ventilated plastic containers containing 60mL dechlorinated tap water. Next day, the individuals were transferred into fresh, ventilated plastic containers (185 x115 x 95mm), lined with moist tissue and provided with plastic piping as shelter. At 7 and 14-days post inoculation, swab samples were taken from the inoculated individuals and infection loads were determined by qPCR. As soon as one of the source-animals was *Bd* or *Bsal* positive, the transmission experiment was started. The infected source-animal was manipulated using gloved hands, mimicking the same actions as when taking a skin swab in the field. Then, the infected individual was returned to its container. One minute later (replicating the minimal time to catch a new individual in the field), a naïve contact-animal was manipulated following the same procedure, still wearing the same pair of gloves. These actions were repeated for each of the source-animals (n = 10) once infection was confirmed by qPCR. The same actions as described above were performed in the non-gloved group, except that the infected source-animals were manipulated with bare hands that had been washed with neutral soap (Soft Care™, Diversey) and thoroughly rinsed with water. After manipulation of each contact animal, hands were disinfected using Disolol^®^ [28]. Between each replicate, a minimal pause of 2 hours was respected, as multiple disinfection cleansing of the hands might negatively affect the antimicrobial function of the amphibian skin [14]. After the transmission experiment, the contact-animals were housed individually as described earlier and infection loads on the skin were monitored by swab sampling and qPCR at a 7-day interval, up to 5 weeks for *Bd*-exposed midwife toads and up to 11 weeks for *Bsal*-exposed Alpine newts.

Once all source and contact-animals had been exposed to *Bd* or *Bsal*, they were checked daily for the presence of clinical signs (excessive skin shedding, lethargy, ulcerations). The infected source-animals received antifungal treatment immediately after the transmission assay, in order to prevent development of more severe illness and possibly death from chytridiomycosis. Contact-animals were treated as soon as they tested positive for infection. For treatment, *Bsal*-infected Alpine newts were housed at 25°C for 10 days [29], while *Bd-*infected midwife toads were sprayed daily with a voriconazole suspension (1,25mg/L water) during 7 days [30]. After treatment, the individuals were followed up by skin swab and qPCR for 4 subsequent weeks. After testing negative in each round of qPCR, the amphibians continued to be housed for future experiments.

Statistical analyses were carried out as described earlier. Transmission rates of *Bd* and *Bsal* were compared between treatments (gloves versus bare-hands) in a non-parametric Mann-Whitney U test and log odds and transmission probability among newts exposed to source animals with varying *Bsal* infection loads was analysed also using binomial logistic regression and a one-tailed Fisher Exact Test using R statistical software [20].

### Identification of fungicidal compounds in rinse water from nitrile gloves

According to the manufacturers’ information, the nitrile gloves we assayed throughout this study are covered at the outside with a coagulant providing the gloves a firm grip (and not intended to have an antimicrobial action). This coagulant layer consists of more than 20 compounds with calcium nitrate, a polystearate compound and water as main compounds. In first instance, we sought to confirm the presence of calcium nitrate and polystearate in the rinse water samples and their antifungal activity. Additionally, we searched for other manufacturing residues present in the rinse water samples that instantly kill chytrid fungi.

### Quantification of calcium nitrate in the rinse water

This assay was performed to determine whether concentrations of calcium nitrate on gloves are sufficient to indeed kill spores. Using a semi-micro colorimetric assay (nitrite/nitrate assay, Roche Life Science, Vilvoorde, Belgium) nitrate NO_3_-N concentrations in the rinse water samples were quantified following the manufacturers guidelines. Because of the detection limit of the assay, samples were diluted 1:500 in ultrapure and NO_3_-N-free distilled water to yield NO_3_–N concentrations between 0.05ng/μL and 5.00ng/μL. NO_3_-N is reduced to nitrite (NO_2_–N) by reduced nicotinamide adenine dinucleotide phosphate (NADPH) in the presence of the enzyme nitrate reductase (NR). The formed NO_2_–N reacts with sulphanilamide and the N-(1-naphthyl)-ethylene-diamine dihydrochloride to give a red-violet diazo dye. The absorbance of the diazo-dye was measured in polystyrene semi-micro cuvettes (Greiner Bio-One, Vilvoorde, Belgium) at 540nm, using a spectrophotometer (Ultrospec III, Pharmacia LKB Biotechnology, Biochrom Ltd, Cambridge, UK). The assay was carried out in duplicate.

The absorbance of known nitrite standards (a known concentration of nitrite used to help determine the concentration of nitrite in a sample of unknown nitrite concentration) ranging from 0.05ng/μL to 5.00ng/μL was measured. Then the same concentration of nitrite was added to samples of unknown nitrate concentrations. The difference in the detected absorbance of the mixed (nitrite + nitrate/unknown) samples and the absorbance of the nitrite (known) samples revealed the absorbance of nitrate in the unknown samples. A calibration curve was created based on the concentrations and corresponding absorbances of nitrate. To determine the nitrate concentration of the unknown samples, their absorbance was plotted on the calibration curve and the points of intersection for each sample was identified as the concentration of each sample. The final concentration of each sample was calculated and the mean and standard deviation of the final concentrations of both measurements of concentration of calcium nitrate of glove batches 1–3 were also calculated.

### Analysis of fungicidal compounds by UHPLC-HRMS

This assay was performed to identify the compounds present in the rinse water which were responsible for the decrease in motility, viability and growth in *Bd* and *Bsal* zoospores. To facilitate identification of the fungicidal component(s) present in the rinse water, the inhibitory effect of rinse water collected from several types and brands of nitrile gloves, was evaluated as described earlier (inhibition of motility, vitality and growth). A total of 7 glove rinse water samples (see S2 Table) were included in the analysis. The HPLC water used to rinse off the gloves, was used as negative control. Identification of the fungicidal compounds and their metabolites was achieved using ultra-high-pressure liquid chromatography coupled with high resolution/high accuracy Orbitrap® mass spectrometry (UHPLC-HRMS) [31] (S1 Fig).

The LC-system consisted of an Accela UHPLC pumping system, coupled to an Accela autosampler and degasser (Thermo Fisher Scientific, San José, CA, USA). Chromatographic separation was achieved by reversed phase chromatography and gradient elution. The different rinse water samples were separated over a BEH C18 column (1.7μm, 100mm x 2.1mm; Waters Corporation, MA, USA), kept at 30°C, running on a 2/98 solvent combination of methanol (A) and 0.5% acetic acid (B) at a flow rate of 0.3mL minute^-1^. The linear gradient was passed off as follows for 13 minutes: A/B at 2/98 for 0.5 minute, increasing to 100/0 for 9 minutes and finally re-equilibrating at 2/98 for 1.50 minutes at initial conditions, before each run.

HRMS analysis was performed on an Exactive^TM^ benchtop mass spectrometer (Thermo Fisher Scientific) fitted with a heated electrospray ionisation probe (HESI) operating in positive and negative ion mode. Applied working conditions were as follows: source voltage at 4.50 kV; capillary voltage at -30 V; tube lens voltage at -95 V; vaporizer and capillary temperatures at 200 and 250°C; sheath and auxiliary gas at 25 and 5 arbitrary units (a.u.). A balanced automatic gain control (AGC) target, an ultra-high mass resolution of 50,000 full width at half maximum resolution (FWHM) at 2Hz (2 scans per second) and a scan range of *m/z* 100–2000 were chosen. The maximum injection time was 500ms. Instrument control and data processing were carried out by the Xcalibur 2.1 software (Thermo Fisher Scientific). SIEVE^TM^ software (version 2.2, Thermo Fisher Scientific) was used for selection of compounds with statistically significant intersample differences in abundance.

Several different types of gloves were included in a preliminary MS-screening. Validation and further testing were performed with the Ecoshield gloves because they were most abundant in the lab and in vitro/ in vivo tests were performed using this type of glove. UHPLC-HRMS data were filtered by selecting out (i) compounds present in the rinse water collected from all types of gloves exhibiting fungicidal activity and absent in the control samples, (ii) compounds with a coefficient of variation (CV) for peak intensity < 30%, for each type of gloves tested, and (iii) compounds for which the experimentally determined mass (*m/z*) yielded a chromatogram characterized by a single, clear and steep peak. For a presumptive identification of the remaining unknown compounds, SIEVE^TM^ software allowed us to retrieve molecules (including appropriate adducts: +nH, +K, +Na, +NH_4_) with a mass deviation less than 10 parts per million (ppm), by performing exhaustive searching through the ChemSpider database [32]. Mass deviations were defined as 10^6^ x ☯(measured mass—theoretical mass)/theoretical mass]. To confirm the structure of the purified compounds, analytical standards were purchased from Sigma-Aldrich (St. Louis, USA) and were processed as were described above. Only compounds for which the retention time (i.e. the time interval between sample injection and the maximum of the peak, which is characteristic of the identity of the component under the operating conditions) and measured elemental composition corresponded with those found for their analytical standard, were retained.

### Quantification of calcium lactate in the rinse water

Quantification of calcium lactate in the rinse water samples was performed using the standard addition approach [33]. Rinse water samples were diluted and each sample was divided in 2 aliquots. One group of aliquots (the known) were spiked with 10μL (*V*_A_) of a 20ng/μL calcium lactate solution (*ρ*_A_) to a final concentration 1ng/μL. The other aliquots (the unknown) were reconstituted in a 50/50 mixture of 0.5% acetic acid and methanol. UHPLC-HRMS analysis of the spiked aliquots resulted in an area ratio of *χ*_known_ while the non-spiked/unknown resulted in an area ratio of *χ*_unknown._ Using the following formula, the unknown concentration (C _unknown_) of the analyte in the matrix was calculated:

$$C_{\mathrm{unknown}}=\chi_{\mathrm{unknown}}\;\rho_{A}\;V_{A}/(\chi_{\mathrm{known}}‐\chi_{\mathrm{unknown}})$$

In this formula, *C* and *ρ* are concentrations, *χ* is the area ratio, *V* is the volume and A is the identified analyte (calcium lactate).

### Evaluation of the antifungal effect of rinse water compounds *in vitro*

This assay was performed to determine whether the combination of compounds in the rinse water created a synergistic effect on the decrease of motility and viability of *Bd* and *Bsal* zoospores. Calcium lactate and calcium nitrate were diluted in HPLC-water to stock solutions of 100000ng/μL, respectively and were diluted to various concentrations. To each well of a 96-well plate 50μL of a calcium lactate or calcium nitrate dilution was added to 50μL of a zoospore suspension in water, containing approximately 10^4^ *Bd* or *Bsal* zoospores. The final assay concentrations of calcium lactate ranged from 250 to 40000ng/μL, and for calcium nitrate from 500 to 100000ng/μL. To the control wells equal amounts of *Bd* or *Bsal* zoospores were added to water. The effect on motility and viability of *Bd* and *Bsal* zoospores was assessed approximately 15 seconds after addition of calcium lactate and calcium nitrate to the zoospore suspensions, using an inverted microscope. Ten gloves were selected from each of three batches of gloves (30 gloves total) to collect rinse water from each batch. The samples were plated in duplicate wells and the assay was performed in triplicate (3 independent assays) for each pathogen.

Subsequently, we examined whether exposure of *Bd* or *Bsal* to both calcium lactate and nitrate resulted in increased killing. To each well of a 96-well plate, 50μL of a calcium lactate and a calcium nitrate dilution was added to 100μL of a zoospore suspension (containing approximately 2x 10^4^) *Bd* or *Bsal* zoospores. Because the amounts of calcium nitrate found in the rinse water were twice as high as those found for calcium lactate, calcium lactate and calcium nitrate were combined in a 1:2 ratio. Final assay concentrations for calcium lactate were 2000, 4000, 8000, 16000, and 32000ng/μL and for calcium nitrate 4000, 8000, 16000, 32000 and 64000ng/μL. The assay was carried out in triplicate.

## Results

### Nitrile glove coagulant instantly kills 99% of *Bd* and *Bsal zoospores*

Upon addition of *Bd* or *Bsal* zoospores to the rinse water, zoospore motility decreased instantly and within less than a minute complete immobility of all zoospores was achieved. The mean time (s) ± standard error (SE) between addition of live motile zoospores to the coagulant suspensions and complete zoospore immobility is presented in Table 1. The rinse water inhibited *Bd* zoospores faster than those of *Bsal* (17–23 ± 3–5 seconds in *Bd*, compared to 39–82 ± 13–20 seconds in *Bsal* in three glove batches). The effect of the rinse water on the zoospores varied according to the different samples tested. In particular, when zoospores were added to coagulant from batch 1, lysis of the zoospores was observed.

**Table 1:** Time to immobility of *Batrachochytrium spp*. zoospores in rinse water from gloves.

Glove batch	Time (s) to immobilize *Bd*	Time (s) to immobilize *Bsal*
1	23±5	82±20
2	19±4	40±8
3	17±3	39±13

The mean time (s) ± standard error (SE) before live motile zoospores became immotile after exposure to rinse water (from 3 different batches of nitrile gloves) is presented. The assay was carried out in triplicate.

*Bd* and *Bsal* zoospores incubated in a 1:1 mixture of concentrated TGhL-broth and rinse water stayed motile up to 1 hour. In the course of the following week, no growth was observed. Also here, lysis of zoospores exposed to rinse water from batch 1 was observed. In the control wells, zoospores retained motility for up to 24 hours and mature zoosporangia appeared after 4 days’ incubation.

As quantified by EMA-qPCR, exposure to rinse water caused a significant reduction in *Bd* (3.27) and *Bsal* (2.63) viable zoospores compared to the reduction in viable zoospores caused by water (*Bd* 0.5 and *Bsal* 0.49) (p≤0.05). Also here, the fungicidal effect of the rinse water on *Bd* was more pronounced than on *Bsal*, with on average a 2000-fold reduction in viability for *Bd* and a 300-fold reduction for *Bsal* (Table 2). Exposure to rinse water killed *Bd* more efficiently than when heating spores at 85°C for 15 minutes (as done for the positive controls). Conversely, heat treatment killed *Bsal* zoospores more efficiently than exposure to rinse water.

**Table 2:** Effect of gloves coagulant on the viability of *Batrachochytrium* zoospores.

Species	Treatment	Reduction in viability (log)	Remaining viability (%)
*Bd*	gloves	3.20 ± 0,29	0.07 ± 0,04
	heat	2.12 ± 0,39	0.96 ± 0,57
*Bsal*	gloves	2.41 ± 0,25	0.42 ± 0,21
	heat	3.43 ± 0,25	0.04 ± 0,02

Summary of the overall inhibitory effect of coagulant suspensions on the viability of *Bd* and *Bsal* zoospores compared to heat-treatment (15 min at 85°C). Results are presented as mean values ± standard error (SE), n = 9

### Nitrile gloves reduce the risk of transmission of *Bd* and *Bsal*

Handling infected Alpine newts using nitrile gloves did not result in transmission of *Bsal* to naive newts. However, the bare-handed manipulation of newts resulted in transmission to 4 out of 10 contact animals. A one-tailed Fisher Exact test demonstrated an association between the bare-handed manipulation of newts and subsequent *Bsal* infection with a statistical significance of *p* = 0.04. Transmission only occurred when handling source-animals with infection loads exceeding 8000 genomic equivalents (GE) of *Bsal* per swab. A binomial regression analysis demonstrated that each unit increase in GE increased the log odds of becoming infected with *Bsal* by 0.00084.

On highly infected source-animals from which transmission was established, mean *Bsal* infection loads of 54022 ± 36375 GEs per swab were found. Clinical signs that occurred in these highly infected individuals were apathy, and severe skin shedding. The time between transmission and build-up of the infection in the contact-animals to qPCR detectable levels varied: in 2 individuals, infection could be detected after 7 days, in another individual after 14 days and in the final individual only after 28 days.

*Bd* was not transmitted to naïve Midwife Toads regardless of manipulation by bare hands or wearing nitrile gloves. Overall, mean *Bd* infection loads of 3897 ± 3154 GE per swab were found on the-source animals. All infected individuals were treated successfully.

### Calcium lactate and nitrate on nitrile gloves kill chytrid zoospores

Upon UHPLC-HRMS analyses, one of the unknown fungicidal compounds was positively identified as calcium lactate in glove batches 1–3 (S1 Table) with an *m/z* ≈ 219.01725 and retention time of 0.65 min coinciding with the calcium lactate standard and thus confirming identification at the Tier 1 level [34] (S1 Fig). It was found highly abundant in the rinse water samples, and its concentrations varied between the different batches of gloves tested. The average concentration ± standard error (SE) of calcium lactate in glove coagulant was 748 ± 578ng/μL (Table 3). The average amount ± SE of calcium lactate on a single glove was estimated at 164000 ± 127000 ng (Table 4).

**Table 3:** Mean concentrations of calcium nitrate and calcium lactate in rinse water from nitrile gloves (n = 2).

Rinse water sample	calcium nitrate (ng/μL; (mean ± SE)	calcium lactate (ng/μL; mean ± SE)
**Batch 1**	737.50 ± 194. 45	350.24 ± 48.49
**Batch 2**	1672.50 ± 60.10	483.57 ± 77.46
**Batch 3**	2612.50 ± 887.42	1412.14 ± 557.68
**mean**	1674.17± 937.50	748.65 ± 578.46

**Table 4:** Calcium lactate and calcium nitrate minimum lethal concentration and total quantities on one glove.

Compound	Concentration killing 100% chytrid spores		Total amount of compound on a single glove
	*Bd*	*Bsal*	
Calcium lactate	15 000ng/μL	15 000ng/μL	164 000 ± 127 000ng
Calcium nitrate	8000ng/μL	16 000ng/μL	167 000 ±93 000ng

Concentrations of calcium lactate and calcium nitrate that have produced 100% *Bd* and *Bsal* mortality and the total amount of calcium lactate and calcium nitrate detected on a single glove.

Based on the glove manufacturers’ information sheets, calcium nitrate was also one of the compounds used in the manufacture of the gloves used in this experiment. However, calcium nitrate could not be detected using UHPLC-HRMS as it did not ionize to a detectable level using ESI, as confirmed upon infusion of the standard directly into the ESI source. Also, stearate-like compounds were not identified by UHPLC-HRMS analyses.

Using a semi-micro colorimetric assay, mean nitrate concentrations ± SE of 1674 ± 932ng/μL were found in the rinse water samples. The average amount of nitrate ± SE on a single glove was estimated to 167000 ± 93000ng (Table 4).

*In vitro*, concentrations of 15000ng/μL calcium lactate in water, and higher, immediately killed *Bd* and *Bsal* zoospores. For calcium nitrate, concentrations of 8000ng/μL in water, and higher, immediately killed *Bsal* zoospores. For *Bd*, nitrate concentrations of 16 000ng/μL were required for immediate killing. Combination of both residues, at lower concentrations than those mentioned above, did not result in a synergistic antifungal effect.

## Discussion

The one-tailed Fisher Exact analysis established that bare-handed manipulation promoted *Bsal* transmission from highly infected newts to naïve contact newts. Wearing a single pair of gloves per animal handled in the field, is optimal in preventing transmission of infections during field activities with amphibians, but its implementation may be hampered by practical and financial constraints. Notwithstanding, we here demonstrate that halving the number of gloves used in the field may still be sufficient to prevent chytrid transmission.

Our results show that nitrile gloves exert a fungicidal action on both *Bd* and *Bsal*. Under controlled laboratory conditions, rinse water from nitrile gloves instantly killed *Bd* to a higher extent than *Bsal* zoospores and also reduced *Bd* motility, two to approximately four times faster than that of *Bsal*. This may be due to differences in both fungi’s cell membrane architecture and/or physiology [8]. The fungal cell wall is largely composed of chitin and constitutes a significant blockade against antifungal agents. *Bd* and *Bsal* possess a chitin-binding module, CBM18, which is suspected to confer the ability to survive on amphibian hosts. As, other pathogenic fungi’s CBMs compete with chitinases, by binding to their own chitin in their cell wall, it has been suggested that *Bd’*s CBM18 provide protection against amphibian host chitinases [28]. Fungal catalases and peroxidases are also able to degrade certain antifungal compounds. It is worth noting that catalase potency in *Bsal* is 3 times higher than in *Bd*. According to Farrer et al [35], the pathogenicity of *Bd* and *Bsal* compared to their saprophytic family members is linked to the larger group of proteases and cell wall gene families,

Other aspects of the fungi’s cell wall composition and physiology also affect their susceptibility to fungicidal compounds. When zoospores which are surrounded by plasma membranes retract their flagella to form zoosporangia, they become surrounded by a thick cell wall. This cell wall may limit permeability or become impermeable to certain antifungal compounds. Also, the clustering of zoosporangia may insulate or buffer the more centrally located zoosporangia from exposure to the antifungal compounds and limit contact, compared to the more peripherally located zoosporangia. These factors contribute to and may explain the need for prolonged periods of exposure to and higher concentrations of antifungal chemicals to achieve complete mortality of *Bsal* compared to *Bd* [28].

Coating compounds used on the outsides of the gloves were shown to exert antifungal activity against both *Bd* and *Bsal*. One of two compounds identified was calcium nitrate. During the manufacturing process, hand-shaped ceramic moulds are dipped into a solution of a calcium salt (e.g. calcium nitrate) in water. A thin film of calcium nitrate remains on the mould and promotes a uniform distribution and coagulation of nitrile or latex around the mould In addition, the coagulant solution sometimes contains a lubrication agent, such as a stearate compound to facilitate stripping of the gloves from the mould at the end of the production line [36–38]. Mean NO_3_-N quantities and concentrations on the outside of the nitrile gloves (167 000 ± 93000ng) we used and in the derived rinse water samples were high (1674 ± 932ng/μL). We demonstrated that 8 000ng/μL and 16 000ng/μL of nitrate are capable of quickly killing *Bd* and *Bsal* zoospores respectively. Therefore, the quantity contained on a single glove was sufficient to kill *Bd* and *Bsal* zoospores instantly, while the quantity in the rinse water was insufficient, which bears out the incomplete killing of *Bsal* and *Bd* zoospores in the viability and growth assay.

However, such concentrations correspond to toxic concentrations for amphibian larvae. Concentrations of 100ng/μL NO_3_-N cause, at least during long-term exposure, increased larval mortality and reduced growth of developing tadpoles of the southern leopard frog (*Rana sphenocephalica*) [39] and Chinese toads (*Bufo gargarizans*) [40]. Also NO_3_-N concentrations of 500ng/μL induce 100% larval mortality in common frogs (*Rana temporaria*) [41]. The adverse effects of NO_3_-N exposure in amphibians however, may vary among species [42] and among populations of the same species [43]. High nitrate concentrations may explain well known negative effects of nitrile, latex and vinyl gloves on tadpoles, even after short-term exposures during routine handling [18] or after exposure to water in which gloves had been soaked [17, 19]. As a consequence, when working with amphibian larvae, it is important to verify NO_3_-N levels on the gloves that are used. This can be done by simply wetting the gloves (with NO_3_-N-free water) and applying a dip-stick used for assessing water quality in aquaria and ponds.

Rinsing gloves in water reduces NO_3_-N concentrations and its toxic effects [18, 19]. However, such rinsing is likely to remove the antifungal properties of the gloves. It must be acknowledged that while handling amphibians in the field, the gloves will become wet, thus, also reducing their antifungal properties. However, not necessarily to the degree as when intentionally washing or rinsing gloves in tap or pond water for the purpose of removing potentially toxic residues. When washing gloves on site, it is advised not to discard the rinse water in fresh water [16].

The other antifungal compound identified at a mean concentration of 748ng/μL in the rinse water and mean quantity of 164 000ng on a single glove, was calcium lactate, which was confirmed using UHPLC-HRMS (Tier 1). Many different types of gloves were included in a preliminary mass spectrometry screening and calcium lactate was found in all nitrile gloves assayed as well as in the latex gloves. This underscores the importance of exercising great care when manipulating pre-metamorphic amphibians using any type of gloves. Validation and further testing was performed with the Ecoshield gloves because they were the most abundant in the lab, and all *in vivo* and *in vitro* tests were performed using these gloves.

Neither of these compounds occur naturally in amphibian habitats or the environment in general. Calcium lactate is often used in the detergent industry as a foaming agent and is known to have antimicrobial properties. The analytical standard and three batches of gloves all displayed identical *m/z* (S1 Fig) (< 1 ppm mass deviation) and retention times. Calcium lactate at 2–3% is often used to decrease microbial spoilage of fresh cut fruits [44, 45] or as carcass decontaminant to reduce microbial contamination with coliform bactpotential eria (*Escherichia coli*) and *Listeria monocytogenes* [46]. *In vitro*, *Bd* and *Bsal* were instantly killed by calcium lactate at a 1.5% solution (or 15000ng/μL). Therefore the quantity contained on a single glove was sufficient to instantly kill *Bd* and *Bsal* while the concentration in the rinse water was insufficient, which bears out the incomplete killing of *Bd* and *Bsal* zoospores in the viability and growth assay.

Calcium nitrate has been used to provide supplemental nitrate or calcium to plants. Solutions of calcium nitrate are added to irrigation water and foliar and fruit sprays to combat deficiencies which lead to decreased crop production and quality. Some research has demonstrated that plants with sufficient calcium are able to fight both biotic and abiotic stressors such as fungal diseases, heat and cold [47]. It has also been used in calcium therapy, in manufacturing as a food thickener or stabilizer and as a feed supplement [48]. In the mitigation toolbox, chemical environmental manipulation has been considered and continues to be considered, a viable short-term mitigation tool against chytridiomycosis. These *in-situ* chemical interventions can be implemented to reduce the viability and spread of the pathogen in the environment [49]. However, upon contemplation of utilizing these two compounds in conservation applications, especially in concentrations high enough to kill *Bd* and *Bsal*, one has to consider the effect on other organisms upon which these compounds may exhibit deleterious effects. As both compounds are salts, high concentrations in the environment may have negative impacts on amphibians (both juveniles and adults) and other organisms [50–52].

Several other compounds were detected using UHPLC-HRMS but did not exactly match with hypothesised analytical standards (S1 Fig) and thus failed to return an exact identification. These unidentified compounds may also contribute to the killing effect of nitrile gloves or work synergistically [53] with calcium lactate and calcium nitrate or potentiate them or other unidentified compounds. Blooi et al. [53] describe successfully utilizing the synergy between voriconazole, polymyxin E and temperature to treat *Bsal* in salamanders.

## Conclusions

While the single use of nitrile gloves should be advocated to handle amphibians, and gloves should always be changed between populations, the repeated use of the same pair of gloves should be preferred above handling amphibians bare handed, since the gloves’ antifungal properties reduce disease transmission, at least in the case of amphibian chytrid fungi. Since rinsing (advised to reduce toxicity for tadpoles), is likely to remove the antifungal effect, the recommendation of using one pair of gloves within a population repeatedly, cannot be transferred to the manipulation of tadpoles.

## Supporting information

S1 Fig HRMS-spectra and chromatograms of the unknown fungicidal compound (A) and the calcium lactate analytical standard (B). In the MS-spectra (at the left) the associated ☯M + H]+ ion (m/z) is indicated. RT: retention time; AA: approximate area, which is proportional to the abundance of the component in the sample. The concentration of the calcium lactate standard injected was 1 ng/μL.
(TIF)

S1 Table Batch data of the nitrile gloves assayed. (PDF)

S2 Table Overview of the different gloves types that were included in mass spectrometric analysis. (PDF)
