# Supplementary figures and images for: Instant killing of pathogenic chytrid fungi by disposable nitrile gloves prevents disease transmission between amphibians
Source: PLoS One. 2020 Oct 29;15(10):e0241048. doi: 10.1371/journal.pone.0241048 (PMC7595420; doi:10.1371/journal.pone.0241048)

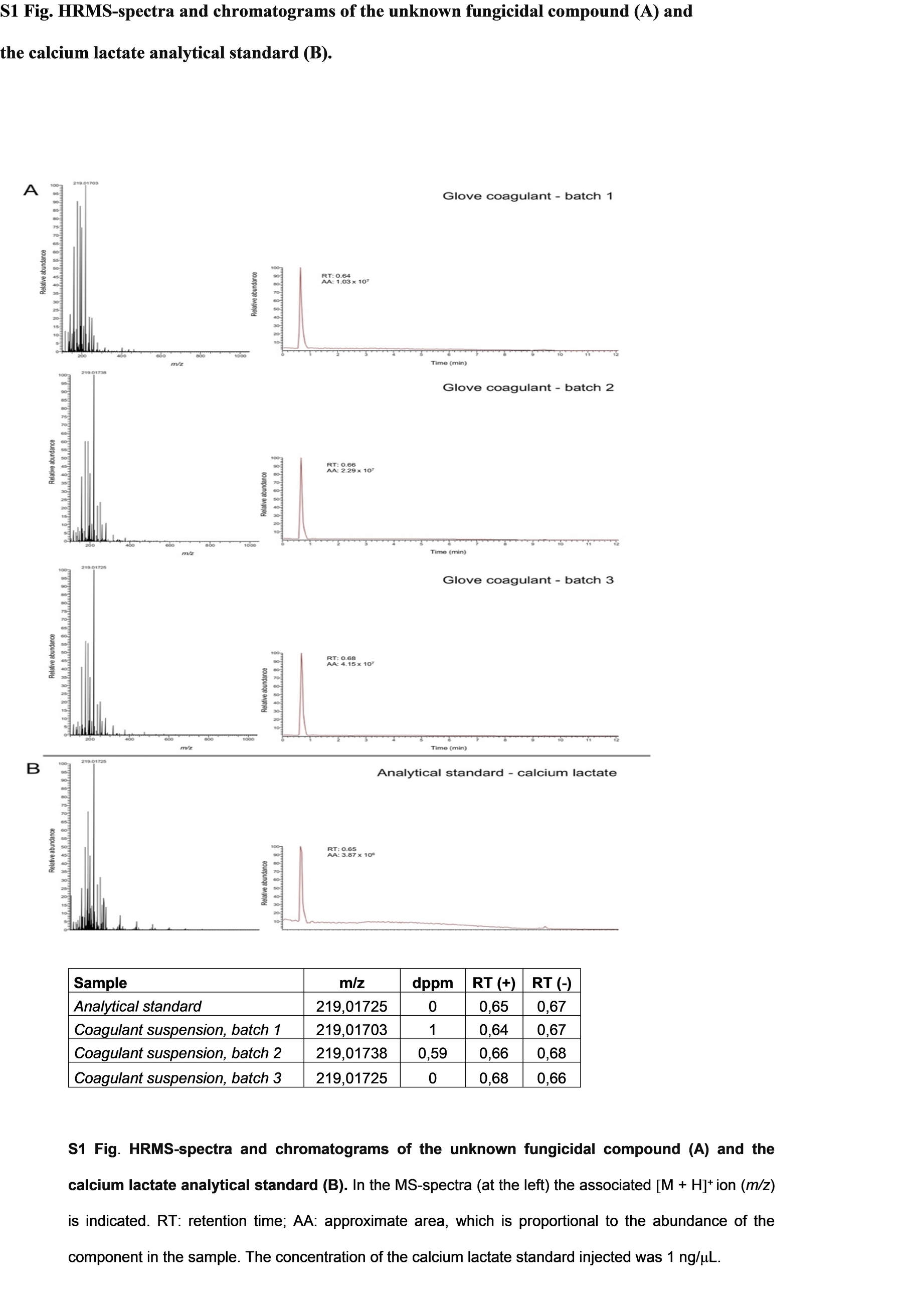

Supplement: S1 Fig — HRMS-spectra and chromatograms of the unknown fungicidal compound (A) and the calcium lactate analytical standard (B). In the MS-spectra (at the left) the associated ☯M + H]+ ion (m/z) is indicated. RT: retention time; AA: approximate area, which is proportional to the abundance of the component in the sample. The concentration of the calcium lactate standard injected was 1 ng/μL. (TIF) [file pone.0241048.s001.tif]
