# Supplementary material for: Instant killing of pathogenic chytrid fungi by disposable nitrile gloves prevents disease transmission between amphibians
Source: PLoS One. 2020 Oct 29;15(10):e0241048. doi: 10.1371/journal.pone.0241048 (PMC7595420; doi:10.1371/journal.pone.0241048)
Supplement: S1 Table — (PDF) [file pone.0241048.s002.pdf]

S1 Table. Batch data of the nitrile gloves assayed.

Batch	Date of manufacture	Expiry date	Batch number
1	June 2012	May 2017	2F10789B
2	February 2014	January 2019	4B05227B
3	January 2014	December 2018	4A20183B
