# Supplementary material for: Instant killing of pathogenic chytrid fungi by disposable nitrile gloves prevents disease transmission between amphibians
Source: PLoS One. 2020 Oct 29;15(10):e0241048. doi: 10.1371/journal.pone.0241048 (PMC7595420; doi:10.1371/journal.pone.0241048)
Supplement: S2 Table — (PDF) [file pone.0241048.s003.pdf]

S2 Table. Overview of the different gloves types that were included in mass spectrometric analysis.

Brand	Glove type	Batch	Powdered/Non-Powdered	Inhibition of *Bd/Bsal*
Ecoshield (eco-nitrile PF 250)	nitrile, white	2F10789B	NP	immediate
Ecoshield (eco-nitrile PF 250)	nitrile, white	4B05227B	NP	immediate
Ecoshield (eco-nitrile PF 250)	nitrile, white	4A20183B	NP	immediate
Ecoshield (eco-nitrile PF 250; ref. 625122)	nitrile, green	5G061256B	NP	immediate
Kimtech (Kimberly-Clark; ref. 90626)	nitrile	700515/SM5127ZZZ-33AX	NP	immediate
Kleenguard (Kimberly-Clark)	nitrile	SM52541xx	NP	immediate
Kleenguard (Kimberly-Clark; ref. 57273)	latex	SH5253306	P	delayed
